# Impact of New Water Sources on the Overall Water Network: An Optimisation Approach

**DOI:** 10.1155/2014/958650

**Published:** 2014-10-29

**Authors:** Godfrey Chagwiza, Brian C. Jones, Senelani D. Hove-Musekwa

**Affiliations:** Department of Applied Mathematics, National University of Science & Technology, P.O. Box AC939 Ascot, Bulawayo, Zimbabwe

## Abstract

A mathematical programming problem is formulated for a water network with new water sources included. Salinity and water hardness are considered in the model, which is later solved using the Max-Min Ant System (MMAS) to assess the impact of new water sources on the total cost of the existing network. It is efficient to include new water sources if the distances to them are short or if there is a high penalty associated with failure to meet demand. Desalination unit costs also significantly affect the decision whether to install new water sources into the existing network while softening costs are generally negligible in making such decisions. Experimental results show that, in the example considered, it is efficient to reduce number of desalination plants to remain with one central plant. The Max-Min Ant System algorithm seems to be an effective method as shown by least computational time as compared to the commercial solver Cplex.

## 1. Introduction

Water source choice depends on water availability, cost of operation and development, quality of water, and adequacy. It is important to evaluate all alternative water sources to ascertain the cost associated with each source. Economic, environmental, engineering, and energy factors must also be considered when choosing the source of water. In semiarid countries, the allocation of water is a particular challenge as there are high costs associated with construction of water distribution systems (WDSs).

In recent years, several researchers have focused on the development of mathematical techniques to minimise the costs associated with constructing water distribution infrastructure. Research has been carried out on the implementation of evolutionary algorithms (EA) in various fields. Algorithms that were used include genetic [1–4], Ant Colony Optimisation [5], and simulated annealing [6]. The genetic algorithm was also applied in the autocalibration of a chlorine transport model [7]. Ant System (AS) and Max-Min Ant System (MMAS) were applied to three WDS case studies [8]. In this research, a model to predict the impact of new water sources is developed and the Max-Min Ant System (MMAS) is used to find the minimum cost.

The remainder of the paper is organised as follows. In Section 2, we review the Ant Colony algorithms and application of the MMAS algorithm. In Section 3, we present a mathematical programming model of a water network with inclusion of the new water sources.  Section 4 gives a numeric example and experimental application of the MMAS algorithm. In Section 5, we present the experimental results of MMAS.  Section 6 gives the summary of the results and points out new directions for further research.

## 2. Review of Ant Colony Algorithms

Ant Colony algorithms use artificial ants and the problems that are solved by these algorithms belong to shortest path problems that are constrained. The characteristics of such problems are the following: there is a finite set of components, *M* = {*m*_1_, *m*_2_,…, *m*_*l*_}, there is a set of constraints which are defined for the problem, there is a cost associated with each solution, and the problem presents a set of all possible sequences, say Γ and $\tilde{\Gamma }$ set of all feasible sequences, with respect to constraints and the solution $S\in \tilde{\Gamma }$ verifying all requirements [10]. These characteristics hold for all combinatorial optimisation problems. Several algorithms have been developed following Ant Colony metaheuristics for combinatorial optimisation, and among them are Ant Systems [11], Ant Colony System [12], MMAS [13], Best-Worst Ant System [14], and Rank-Best Ant System [15]. Ant Colony Optimisation (ACO) performs well on problems that show correlation between distance to global optima and fitness of the solutions [13]. ACO in recent years has been defined and applied to solve complex combinatorial problems. Based on the conclusions by Cordón et al. [16], we propose to use MMAS, and a brief overview of the algorithm is presented in Section 2.1.

### 2.1. Max-Min Ant System

The MMAS algorithm is an adaptation of the general Ant System, which was developed from the behaviour of ants when searching for food [17]. Ants deposit an aromatic substance, called a pheromone, when finding food. After some time, the pheromone trail disappears if no other ants use the same path, resulting in the more frequented paths retaining a higher intensity of pheromone. The Ant System associates pheromone trails with the solution of combinatorial problems.

Evolutionary algorithms (EAs) converge to suboptimal solutions prematurely and this is a problem, especially in those cases that have a greater emphasis on exploitation [8]. MMAS was developed by Stützle and Hoos in 2000 [13] to overcome this problem. MMAS provides dynamically evolving bounds on the pheromone trail intensities such that the pheromone intensity on all paths is always within a specified limit of the path with the greatest pheromone intensity. All paths have a nontrivial selection probability. MMAS uses upper and lower bounds to ensure that pheromone intensities lie within a given range, which means *τ*_min⁡_(*t*) ≤ *τ*_*ij*_(*t*) ≤ *τ*_max⁡_(*t*). The upper and lower limits are given, respectively,

(1) $$\begin{array}{c} \;\tau_{\max}\left(t\right)=\frac{1}{\left(1-\rho \right)}\left(\frac{1}{f\left(S^{\text{gb}}\left(t-1\right)\right)}\right),\\ \tau_{\min}\left(t\right)=\frac{\tau_{\max}\left(t\right)\left(1-\sqrt[{n}]{P_{\text{best}}}\right)}{\left({\text{NO}}_{\text{avg}}-1\right)\sqrt[{n}]{P_{\text{best}}}}, \end{array}$$

where *τ*_*ij*_(*t*) is the concentration of pheromone associated with edge (*ij*) in iteration *t*, *ρ* is the coefficient representing pheromone persistence so 1 − *ρ* represents the evaporation of trail and 0 ≤ *ρ* ≤ 1, *P*_best_ is the probability that the current global-best path, *S* ^gb^(*t*), will be selected given that all nonglobal-best edges have a pheromone level of *τ*_min⁡_(*t*) and all global-best edges have a pheromone level of *τ*_max⁡_(*t*), *n* is the number of decision points, NO_avg_ is the average number of edges at each decision point, and *f*(·) is the objective function [8].

The best ant that is allowed to add pheromone may be the best iteration. The problem of stagnation is decreased by giving each connection a chance of being chosen. MMAS uses reinitialization of pheromone trails in order to increase evaporation of the solution. MMAS was used to find a general solution method for the multilevel capacitated lot-sizing and scheduling problem [18], in multiobjective problems [19] and in routing problems [20]. The MMAS algorithm can be used to solve NP-hard problems (no algorithm is known to solve the problems in polynomial time [21]) and it incorporates aggressive pheromone update procedures to avoid stagnation. The algorithm was designed to have a long initial exploration phase and a subsequent transition to a strong exploitation phase [22]. MMAS was used for pipe network optimisation of existing water networks and it performed well [23].

We propose to apply the methodology in a relatively new area, in the sense that we are introducing new water sources to the existing network and optimising the network inclusive of the new sources. The resulting problem is more complex compared to optimising an existing network as several solutions exist as to how to layout the pipelines from new sources to the existing network.

## 3. New Water Sources and Pipelines

We can expand the existing water system to supplement demand but the economies of using the existing sources should be evaluated using alternatives.

### 3.1. Water Salinity Consideration

It is important to consider salinity levels from these sources in order to determine the actual costs of supplying the water from these sources. The parameters are defined in Table 1:

(2) $$\begin{array}{c} Q=S_{L_{r}}Q_{L_{r}}+S_{F_{r}}Q_{F_{r}}+S_{G_{r}}Q_{G_{r}}+S_{P_{r}}Q_{P_{r}}. \end{array}$$

We need desalination if the threshold has been surpassed. Thus, the salinity level of untreated water should be higher than that of treated water plus lost water during water treatment. This is to ensure that desalination is effective. Hence,

(3) $$\begin{array}{c} \left(Q_{L_{r}}-Q_{L_{t}}\right)S_{L_{r}}+Q_{L_{t}}S_{L_{t}}\leq Q_{L_{r}}S_{L_{r}},\\ \left(Q_{F_{r}}-Q_{F_{t}}\right)S_{F_{r}}+Q_{F_{t}}S_{F_{t}}\leq Q_{F_{r}}S_{F_{r}},\\ \left(Q_{G_{r}}-Q_{G_{t}}\right)S_{G_{r}}+Q_{G_{t}}S_{G_{t}}\leq Q_{G_{r}}S_{G_{r}},\\ \left(Q_{P_{r}}-Q_{P_{t}}\right)S_{P_{r}}+Q_{P_{t}}S_{P_{t}}\leq Q_{P_{r}}S_{P_{r}}. \end{array}$$

Using (3), if we have *n* sources of *j* type, we get (4), the salinity constraint

(4) $$\begin{array}{c} \left(Q_{n,j}-Q_{n,j}^{tr}\right)S_{n,j}+Q_{n,j}^{tr}S_{n,j}^{tr}\leq Q_{n,j}S_{n,j}. \end{array}$$

Let *S* ^*^ be the required salinity level of water suitable for consumption; then

(5) $$\begin{array}{c} S_{L_{r}}+S_{F_{r}}+S_{G_{r}}+S_{P_{r}}=S^{*}. \end{array}$$

If we are getting water from our local source only, then

(6) $$\begin{array}{c} S_{L_{r}}=S^{*}. \end{array}$$

Using the same concept we used to get (6), it follows that

(7) $$\begin{array}{c} S_{L_{r}}=S_{F_{r}}=S_{G_{r}}=S_{P_{r}}=S^{*}. \end{array}$$

The amount of water supplied for consumption can be calculated as the sum of the differences between quantity of water from each source and quantity of the treated water multiplied by their salinity level. Therefore,

(8) (QLr−QLt)SLr+(QFr−QFt)SFr+(QGr−QGt)SGr  +(QPr−QPt)SPr+QLtSLt+QFtSFt  +QGtSGt+QPtSPt=[QLr+QFr+QGr+QPr]S∗⟹QLtSLt−QLtSLr+QFtSFt−QFtSFr+QGtSGt  −QGtSGr+QPtSPt−QPtSPr  +QLrSLr+QFrSFr+QGrSGr+QPrSPr =QLrS∗+QFrS∗+QGrS∗+QPrS∗⟹QLt(SLt−SLr)+QFt(SFt−SFr)+QGt(SGt−SGr)  +QPt(SPt−SP)+QLrSLr+QFrSFr  +QGrSGr+QPrSPr =QLrS∗+QFrS∗+QGrS∗+QPrS∗.

From (8) it follows that

(9) QLr(SLr−S∗)+QFr(SFr−S∗)+QGr(SGr−S∗)  +QPr(SPr−S∗)=QLt(SLr−SLt)  +QFt(SFr−SFt)+QGt(SGr−SGt)+QPt(SPr−SPt).

Hence, we can determine the actual amount of water from each source that has been desalinated by equating treated to untreated water quantities from the same water source. Thus,

(10) $$\begin{array}{c} Q_{L_{t}}=\frac{Q_{L_{r}}\left(S_{L_{r}}-S^{*}\right)}{S_{L_{r}}-S_{L_{t}}},\\ Q_{F_{t}}=\frac{Q_{F_{r}}\left(S_{F_{r}}-S^{*}\right)}{S_{F_{r}}-S_{F_{t}}},\\ Q_{G_{t}}=\frac{Q_{G_{r}}\left(S_{G_{r}}-S^{*}\right)}{S_{G_{r}}-S_{G_{t}}},\\ Q_{P_{t}}=\frac{Q_{P_{r}}\left(S_{P_{r}}-S^{*}\right)}{S_{P_{r}}-S_{P_{t}}}. \end{array}$$

The cost associated with desalination is important to make a decision on whether to use the new sources. Let *C*_*S*_*tr*__ be the total cost of desalination; then

(11) CStr=PLt(SLr−SLt)+PFt(SFr−SFt) +PGt(SGr−SGt)+PPt(SPr−SPt).

If there are *n* sources of *j* type, we can generalise (11) as

(12) $$\begin{array}{c} D_{\left(n,j\right)}=\overset{N}{\underset{n=1}{\sum }}\;\overset{J}{\underset{j=1}{\sum }}P{\left(S\right)}_{n,j}^{tr}\left(S_{n,j}-S_{n,j}^{tr}\right), \end{array}$$

where *D*_(*n*,*j*)_ is defined in Table 2.

### 3.2. Water Hardness Consideration

Water hardness is important when considering an alternative source of water. Hardness is defined as the amount of minerals found in water and is usually reported as an equivalent quantity of calcium carbonate (CaCO_3_) [24]. Hardness can be reduced by softening. The easiest way to test for water hardness is a lather or frost test. If water is hard, the soap will not lather easily. Hard water causes limescale in kettles and washing machines, but it does not have any health related problems if consumed. For urban use, water hardness is a matter of concern due to the kind of utensils and equipment used that can lather.

The WHO guidelines in 2004 identified that water with a hardness of value 200 mg/L or higher will produce scale and soft water with a value of 100 mg/L or less, will have a low buffering capacity, and will be more corrosive to pipes [24]. The level of CaCO_3_ (mg/L) regarded as hard to extremely hard differs from country to country. The average value is any value above 200 mg/L as CaCO_3_ and this is the value that will be used in this research. We need to soften the water if the level is exceeded. Using the same concept as in (3) and (4) and substituting *S* with *H*, we get the hardness constraint. If we have water from *n* sources of *j* type and taking 200 mg/L as the maximum level, then

(13) $$\begin{array}{c} 100<\overset{N}{\underset{n=1}{\sum }}\;\overset{J}{\underset{j=1}{\sum }}H_{n,j}<200. \end{array}$$

Replacing *P*_(·)_ with *C*_(·)_ and *S*_(·)_ with *H*_(·)_ in (12), we get the cost associated with softening *C*_*H*_*tr*__.

### 3.3. Formulation of the Model

A mixed integer programming problem (MIP) is formulated for the water distribution network considering new water sources. Model parameters (par) and variables (var) are defined in Table 2.

The pumping cost at each new supply node is represented by (15) and it is useful to evaluate the impact of new water sources. It is calculated as the product of pumping cost and additional quantity of water from new water sources. The cost of the new pipes is equal to the sum of distances from new source to reservoir multiplied by the cost per unit length. The distance is calculated as

(14) $$\begin{array}{c} d_{i,j}=\sqrt{{\left(x_{ij}-x_{ji}\right)}^{2}+{\left(y_{ij}-y_{ji}\right)}^{2}}. \end{array}$$

Equation (16) is the cost of additional pipes into the water network. The distance covered by the pipes helps us to determine the number of pipes required. We can calculate the cost as a product of unit cost of pipe of size *s* by the distance covered:

(15) $$\begin{array}{c} U_{\left(n,j\right)}=\overset{N}{\underset{n=1}{\sum }}\;\overset{J}{\underset{j=1}{\sum }}u_{\left(n,j\right)}\times \left(Q_{\left(n,j\right)}^{+}\right), \end{array}$$

(16) $$\begin{array}{c} F_{\left(n,j\right)}=\overset{I}{\underset{i=1}{\sum }}‍\;\overset{J}{\underset{j=1}{\sum }}R_{\left(s\right)}d_{i,j}. \end{array}$$

Although new water sources can be identified, it is imperative to consider the existing water reservoirs' capacity. We can get as much water as possible from new sources but we must also consider limited reservoir capacity. Equation (17) is the objective with the aim of minimising cost of including the new water sources to the existing water network *Z*. The constraint equation (18) shows that the quantity of additional water from new sources, whether desalination or softening has been done, should be less or equal to the additional quantity of water from *n* sources of *j* type added to the existing network.

To ensure that we meet demand, we add (19) to our constraints list which includes additional capacity added to the existing water network. The equations from (20) to (22) are the water desalination and softening constraints. Equation (23) is the storage capacity constraint and the equations from (24) to (27) are sign restriction constraints. Consider

(17) Min⁡     Z=∑n=1N ∑j=1JC(n,j)Q(n,j)        +∑n=1N ∑j=1J(A(n,j)+U(n,j))X(n,j)        +∑n=1N ∑j=1JD(n,j)Y(n,j)        +∑n=1N ∑j=1JE(n,j)W(n,j)        +∑n=1N ∑j=1JF(n,j)G(n,j)subject  to

(18)          (Y(n,j)+W(n,j))Q(n,j)+≤X(n,j)Q(n,j)+,                  ∀n∈N, ∀j∈J

(19)         V≤Q(n,j)+X(n,j)×Q(n,j)+,               ∀n∈N, ∀j∈J

(20)       (Q(n,j)−Qn,jtr)Sn,j+Qn,jtrSn,jtr      ≤Q(n,j)Sn,j,         ∀n∈N,   ∀j∈J

(21)       (Q(n,j)−Qn,jtr)Hn,j+Qn,jtrHn,jtr      ≤Q(n,j)Hn,j,           ∀n∈N, ∀j∈J

(22)        100<∑n=1N ∑j=1JHn,j<200,           ∀n∈N, ∀j∈J

(23)          Q(i,t)−≤Q(i,n,t)≤Q(i,t)+,

(24)          Q(n,j)≥0, ∀n∈N,  ∀j∈J

(25)          X(n,j)∈{0,1}, ∀n∈N,  ∀j∈J

(26)             Y(n,j)∈{0,1}, ∀n∈N,  ∀j∈J

(27)           W(n,j)∈{0,1}, ∀n∈N,  ∀j∈J.

## 4. Model Implementation

The equations from (17) to (27) can be presented as a weighted graph *G* = (*N*, *E*), *G* the graph, *N* set of components, and *E* edges. As highlighted in Section 2, Ant Colony Optimisation algorithms can be used to solve the model. The ability of the MMAS algorithm to model a complex network with large solution space drives the authors to use it in this research. The algorithm requires an initial point or value and lower and upper values to be given. This is ideal for an existing water network since there is a limit on the minimum and maximum quantity of water flowing in the water network to maintain a certain level of pressure and to meet demand. The strength of the algorithm to avoid stagnation was also considered when selecting this algorithm as a method to solve the problem.

The city of Bulawayo in Zimbabwe is facing water allocation challenges and it is imperative to focus on alternative water sources. The city has been hit by a series of drought seasons resulting in the depletion of the current water sources. Nyamandlovu aquifer and groundwater abstraction using boreholes are better alternatives to supplement the current water sources. Desalination and softening were considered. Shortest distance between the new water sources and reservoir was used to compute the cost. The quantity to be abstracted from the aquifer was an estimate obtained from the city of Bulawayo's water engineering department. Costs were also extracted from the city of Bulawayo's master plan.

Three boreholes, in Entumbane, Mpopoma, and the city centre, were considered in this research as sources of ground water. WHO [24, 25] salinity and hardness levels were considered as the levels the water to be supplied to consumers should exhibit.  Table 3 data shows the data of the reservoirs and new water sources. Distance coordinates of each of the areas were obtained from the Zimbabwe National Water Authority (ZINWA). The current water network has six sources, all being local dams. The resultant problem has ten water sources inclusive of the proposed new sources. There are only three water source types considered in this research that is ground, aquifer, and surface water sources. The desalination plant costs were adopted from the International Desalination Association; see [26] for more information. The costs include annualised capital cost and operational (chemicals, energy, total maintenance and labour, and membranes and cartridge filters) costs. The minimum distances from the new sources to the reservoirs are in brackets.

MIDACO version 3.0, an optimisation toolbox which implements MMAS, was used in MATLAB version 7.0.4 to solve the mathematical programming problem. We set *α* = 0.9, *β* = 0.7, and *ρ* = 0.7 using sensitivity analysis of the parameters. Sensitivity analysis of the parameters in emphasizing differences between arcs was done in Excel using the goal seek option of what-if-analysis. The probability was set to 1 and the corresponding values of *α*, *β*, and *ρ* were recorded. Set *m* = 80, *Q* = 2 × 10^3^, and *P*_best_ = 0.01. The current total water allocation cost, including failure to meet demand cost estimated by the city of Bulawayo, is $2300.00 per second. The problem has forty-six constraints, of which thirty-six are surface-treatment plant-reservoir generated, eight are ground water-reservoir generated, and two are aquifer-treatment plant generated. Demand points and daily demand quantities are shown in Table 4.

## 5. Results

The results after implementing the model show that it costs $2107.73 per second, including cost of investment on pipes to meet demand at any given second. Inclusion of new water sources proves to be necessary as the cost of connecting the new water sources to the existing water network is less than the penalty plus water allocation cost. The penalty cost was estimated as that arising from legal and medical costs suggested by Bulawayo Progressive Residence Association for water related cases and illnesses, respectively. The cost might be higher if we were to consider social and welfare, water rationing, and political costs.

Unit cost by capacity predictions of the new desalination plants is used to extrapolate the total maintenance, operational, and capital costs. Capital cost constitutes 37% of unit cost while operational cost constitutes 63% of the unit cost [26]. To achieve this, data was extracted from Global Water Intelligence [26] for 15 small scale desalination plants and fitted to a nonlinear regression model because of the arbitrary relationship between desalination unit cost and capacity of the desalination plant. SPSS package [27] was used to estimate the nonlinear regression model:

(28) $$\begin{array}{c} Y_{i}=\beta_{0}-\beta_{1}\left(1-e^{-(x_{i}/\lambda )}\right)+\epsilon_{i},\;i=1,\ldots ,\lambda , \end{array}$$

where *Y*_*i*_ is unit cost of plant *i*, *x*_*i*_ is capacity of plant *i* and *ϵ*_*i*_ ~ *N*(0, *σ*), and *λ* is the number of plants, and in the case of city of Bulawayo, 3 desalination plants were proposed. The operational cost was calculated as 63% of the unit cost for each new water source.

The results show that it is expensive to build a desalination plant for each new water source as compared to the cost of installing a new water pipeline to a central desalination plant. Although the operational cost was increased from $1220.78 to $1807.00 per second, it was noted that it was less than the total water allocation cost including failure to meet demand cost. Water softening was found not to influence the optimal water network to a large extent; therefore, its impact is negligible in making a decision as to whether to add new water sources to an existing water network. The best network design, which considers distance between the nodes, is presented in Table 5. The corresponding quantity supplied to each reservoir, costs for each reservoir, and the optimal distance new pipes which should cover to reservoirs are also shown. The MMAS algorithm seems to be an efficient tool to find the optimal water network inclusive of new water sources. The time taken to find the optimal solution using MMAS algorithm was 0.935 seconds while solving the same problem using Cplex solver took 1.24 seconds on a PC with the processor speed of 1.30 GHz.

The water authority will cut the total cost by 8.4% after installing the new water sources into the existing network while meeting daily demand and the required pressure. The pumping costs differ between sources due to the distance between source and reservoir and the gradient except for ground water sources where more pumps should be installed to enhance abstraction. Softening and desalination costs also differ due to underlying bedrock and as salinity and hardness levels differ between sources.  Table 6 is a summary of quantity of the water extracted from each source. The grand total is the total of quantities extracted from existing network plus new sources. Comparing this grand total with the daily demand in Table 4 results in the surplus, which is a healthy condition for a water network problem.

## 6. Conclusion

Our results show that desalination unit costs strongly influence the impact of new water sources on the total water allocation cost and hence there is need to reduce the number of desalination plants to cut costs. In our example, water softening is a negligible cost in making a decision to whether new water sources are installed into the existing network. Minimising the distance between new water sources and reservoirs is also a factor to be considered. Judging from the results of this paper and previous applications of MMAS to water distribution systems by Zecchin et al. [8], the MMAS methodology proves to be an effective optimisation algorithm to solve network problems. Impact of new water sources on the existing network's total cost can be found by implementing MMAS. It is therefore concluded that the best water network inclusive of the new sources can be found by using MMAS and it is efficient to include new water sources in a network if the distance between the new source and reservoir is short and if high penalty is associated with failure to meet demand.

## Figures and Tables

**Table 1:** Definitions of raw and treated water quantities, salinity levels, price, and cost of water sources.

Source (*n*)	Water quantity (*Q*)		Salinity level (*S*)		Cost (*P*)	
	Untreated	Treated	Initial	Current	Water source	Average cost of desalination
Local source (*L*)	*Q*_*L*_*r*__	*Q*_*L*_*t*__	*S*_*L*_*r*__	*S*_*L*_*t*__	*P*_*L*_*r*__	*P*_*L*_*t*__
Foreign (*F*)	*Q*_*F*_*r*__	*Q*_*F*_*t*__	*S*_*F*_*r*__	*S*_*F*_*t*__	*P*_*F*_*r*__	*P*_*F*_*t*__
Aquifer (*G*)	*Q*_*G*_*r*__	*Q*_*G*_*t*__	*S*_*G*_*r*__	*S*_*G*_*t*__	*P*_*G*_*r*__	*P*_*G*_*t*__
Rain harvesting (*P*)	*Q*_*P*_*r*__	*Q*_*P*_*t*__	*S*_*P*_*r*__	*S*_*P*_*t*__	*P*_*P*_*r*__	*P*_*P*_*t*__

*n* sources of *j* type	*Q*_*n*,*j*_	*Q*_*n*,*j*_^*tr*^	*S*_*n*,*j*_	*S*_*n*,*j*_^*tr*^	*P*(*S*)_*n*,*j*_	*P*(*S*)_*n*,*j*_^*tr*^

**Table 2:** Definition of model parameters (par) and variables (var).

Name	Definition	Type
*n*	Index denoting number of water sources *n* = 1,…, *N*	par
*j*	Index denoting type of water source, *j* = 1,…, *J*	par
*i*	Index denoting reservoir	par
*t*	Index denoting time period, *t* = 1,…, *T*	par
*A*_(*n*,*j*)_	Annualised capital costs of additional capacity (excluding new pipes costs and pumping costs) from *n* sources of *j* type	par
*C*_(*n*,*j*)_	Existing annualised operating costs of supply costs (operating, maintenance, and environmental costs)	par
*D*_(*n*,*j*)_	Annualised desalination costs from *n* sources of *j* type	par
*E*_(*n*,*j*)_	Annualised softening costs from *n* sources of *j* type	par
*F*_(*n*,*j*)_	Total cost of additional pipes for *n* new water sources of *j* type or investment on pipes cost	par
*Q*_(*i*,*t*)_^−^	Minimum storage capacity of reservoir *i* at period *t*	par
*Q*_(*i*,*n*,*t*)_	Water quantity flowing from *n* sources during period *t* to reservoir *i*	par
*Q*_(*i*,*t*)_^+^	Maximum storage capacity of reservoir *i* at period *t*	par
*Q*_(*n*,*j*)_^+^	Additional quantity of water from *n* new sources of *j* type	par
*R*_(*s*)_	Unit cost of pipe of size *s*	par
*u*_(*n*,*j*)_	Pumping cost per cubic meter	par
*U*_(*n*,*j*)_	The pumping cost of additional water from *n* sources of type *j*	par
*V*	Annualised volume of water demanded in m^3^	par
*G*_(*n*,*j*)_	Quantity of additional pipes required to add *n* new water sources of *j* type	var
*Q*_(*n*,*j*)_	Existing water supply capacity into the water network	var
*X*_(*n*,*j*)_	Indicate whether additional capacity from *n* sources of *j* type is added to the existing water capacity and it is binary	var
*Y*_(*n*,*j*)_	Indicate whether additional capacity from *n* sources of *j* type added is desalinated and it is binary	var
*W*_(*n*,*j*)_	Indicate whether additional capacity from *n* sources of *j* type added is softened and it is binary	var

**Table 3:** Water data (source: city of Bulawayo Master plan).

Node	Distance coordinates		Storage	Supply
	*x*	*y*	Capacity (m^3^/day)	Capacity (m^3^/day)
Tuli Hill	1200	3600	50000	
Criterion	4000	2200	90000	
6J	2000	2300	45000	
Hillside	2300	3200	45000	
Rifle range	750	2400	67500	
Magwegwe number 8	700	3000	108000	
Woodville	900	1200	2250	
Nyamandlovu aquifer	40000	41050		55000
Borehole 1	4000	3907		5000
Borehole 2	3500	3700		6000
Borehole 3	2000	4500		7000

**Table 4:** BCC water consumption levels [9].

Category	Estimated number of consumers	Consumption (m^3^/day)
High density suburbs	94 595	42 000
Low density suburbs	47 297	21 000
Industry	13 966	35 000
Public institutions	12 900	80 000

Total	168758	178000

**Table 5:** Summary of new source to reservoir water allocation.

	Cost ($)	Tuli Hill	Criterion	6J	Hillside	Magwegwe number 8	Rifle range	Woodville
Aquifer	New pipe/m	0.49	0.49	—	—	—	0.34	—
	Pumping/second	1.2	1.2	—	—	—	1.2	—
	Desalination/m^3^	0.20	0.21	—	—	—	0.20	—
	Softening/m^3^	0.10	0.10	—	—	—	0.10	—
	Distance (m)	53925.34	52965.29	—	—	—	55085.25	—

Borehole 1	New pipe/m	—	—	—	—	0.13	—	—
	Pumping/second	—	—	—	—	1.43	—	—
	Desalination/m^3^	—	—	—	—	0.25	—	—
	Softening/m^3^	—	—	—	—	0.15	—	—
	Distance (m)	—	—	—	—	3422.37	—	—

Borehole 2	New pipe/m	—	—	0.12	—	0.12	—	—
	Pumping/second	—	—	1.43	—	1.47	—	—
	Desalination/m^3^	—	—	0.25	—	0.25	—	—
	Softening/m^3^	—	—	0.15	—	0.15	—	—
	Distance (m)	—	—	2051.83	—	2886.17	—	—

Borehole 3	New pipe/m	—	—	—	0.14	—	—	0.15
	Pumping/second	—	—	—	1.43	—	—	1.43
	Desalination/m^3^	—	—	—	0.25	—	—	0.25
	Softening/m^3^	—	—	—	0.15	—	—	0.15
	Distance (m)	—	—	—	1334.17	—	—	1500

Quantity supplied by new source (m^3^/day)								
Aquifer		12000	13000				23000	
Borehole 1						5000		
Borehole 2				4000		2000		
Borehole 3					3800			3200

**Table 6:** Summary of new source and existing abstraction quantities.

	Source name	Quantity supplied (m^3^)
Existing sources	Umzingwane	33151
	Inyankuni	36428
	Ncema complex	60000
	Insiza	45205
Total		** 112475**

New sources	Nyamandlovu aquifer	55000
	Borehole 1	5000
	Borehole 2	6000
	Borehole 3	7000
Total		** 73000**

Grand total		185475

## References

[1] Dandy G. C., Simpson A. R., Murphy L. J. (1996). An improved genetic algorithm for pipe network optimization. *Water Resources Research*.

[2] Savic D. A., Walters G. A. (1997). Genetic algorithms for least-cost design of water distribution networks. *Journal of Water Resources Planning and Management*.

[3] Lippai I., Heaney J. P., Laguna M. (1999). Robust water system design with commercial intelligent search optimizers. *Journal of Computing in Civil Engineering*.

[4] Wu Z. Y., Boulos P. F., Orr C. H., Ro J. J. (2001). Rehabilitation of water distribution system using genetic algorithm. *Journal for American Water Works Association*.

[5] Maier H. R., Simpson A. R., Zecchin A. C. (2003). Ant colony optimization for design of water distribution systems. *Journal of Water Resources Planning and Management*.

[6] da Conceição Cunha M., Sousa J. (1999). Water distribution network design optimization: simulated annealing approach. *Journal of Water Resources Planning and Management*.

[7] Kumar M. S. M., Munallavi G. R. Autocalibration of chlorine transport for steady state distribution by genetic algorithm.

[8] Zecchin A. C., Maier H. R., Simpson A. R., Roberts A. J., Berrisford M. J., Leornard M. Max-min ant system applied to water distribution system optimisation.

[9] Bulawayo City Council (BCC) (2012). Water report for the period January-December 2012. *Annual Water Report*.

[10] Dorigo M., Di Caro G. (1999). New ideas in optimization. *The Ant Colony Optimization Meta-Heuristic*.

[11] Dorigo M., Maniezzo V., Colorni A. (1996). Ant system: Optimization by a colony of cooperating agents. *IEEE Transactions on Systems, Man, and Cybernetics B: Cybernetics*.

[12] Dorigo M., Gambardella L. M. (1997). Ant colony system: a cooperative learning approach to the traveling salesman problem. *IEEE Transactions on Evolutionary Computation*.

[13] Stützle T., Hoos H. H. (2000). Max-min ant system. *Future Generation Computer Systems*.

[14] Cordon O., de Viana I. F., Herrera F., Moreno L.

[15] Bullnheimer B., Hartl R. F., Strauss C. (1999). A new rank based version of the ant system: a computational study. *Central European Journal of Operations Research*.

[16] Cordón O., Herrera F., Stützle T. (2002). A review on the ant colony optimization metaheuristic: basis, models and new trends. *Mathware and Soft Computing*.

[17] Colorni A., Dorigo M., Maffioli M., Maniezzo V., Righini G., Trubian M. (1996). Heuristics from nature for hard combinatorial optimization problems. *International Transactions in Operational Research*.

[18] Almeder C. (2010). A hybrid optimization approach for multi-level capacitated lot-sizing problems. *European Journal of Operational Research*.

[19] Pinto D., Baran B. Solving multiobjective multicast routing problem with a new ant colony optimization approach.

[20] Sodsoon S. (2010). Max-min ant system for location-routing problems. *Suranaree Journal of Science and Technology*.

[21] Garey M. R., Johnson D. S. (1979). *Computer and Intractability: A Guide to the Theory of NP-Completeness*.

[22] Maur M., Lopez-Iba M., Stutzle T. Pre-scheduled and adaptive parameter variation in max-min ant system.

[23] Afshar M. H. (2009). Elitist-mutated ant system versus max-min ant system: application to pipe network optimization problems. *Scientia Iranica*.

[24] WHO (2009). *Calcium and Magnesium in Drinking-Water: Public Health Significance*.

[25] WHO Hardness in drinking water.

[26] Global Water Intelligence (2009). *IDA Desalination Yearbook 2008-2009*.

[27] SPSS (1999). *SPSS Base 10.0 for Windows User's Guide*.

